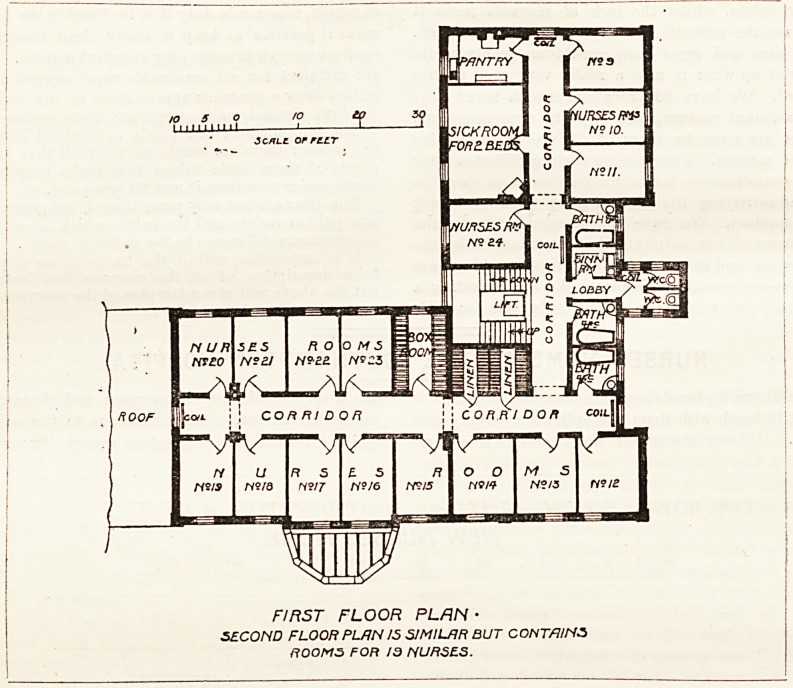# Nurses' Home at the Southampton Hospital

**Published:** 1905-08-05

**Authors:** 


					NURSES' HOME AT THE SOUTHAMPTON HOSPITAL.
The Nurses' Home is placed close to the hospital buildings, is
in fact almost in touch with them, andjadjoins a surgery and
waiting-room. It faces almost due north, and to this front
are a very handsome sitting-room and three other sitting-
rooms for nurses. A corridor runs to the south of these
which gives access to the matron's rooms, two nurses' rooms,
THE ROYAL SOUTH- HANTS and SOUTHAMPTON HOSPITAL
NEW NURSES HOME ? -y
10 S O 10 20 30 90 50 GO JO _
<5colt of i m 11 1 11 I 11 | i i I I I 1 5trJ~
YCLVHG "iP HALL. (ARCHT?)
17 SOUTHAMPTON SI
BLOOMSBURV W.C.
GROUND FLOOR PLAN-
332 THE HOSPITAL. August 5, 1905.
and a bath-room, and opposite to the latter are the
linen cupboards. Between these and the bath-room is
another corridor running north and south and leading to the
assistant matron's rooms, three nurses' rooms, cloak-room,
bath-rooms, and closets, the latter being cut off from the
conditions are as like as may be to a home of the- class to
which the child by birth belongs, and the children associate
freely at school and at play with ordinary children and so
lose the idea of belonging to a class apart. If they are
paupers, at least they need not know it, and thus they escapo.
main bj a cross-ventilated passage. ^ On the first floor are
16 nurses' rooms, linen and box-rooms, bath-rooms, and what
we are glad to notice, a sick-room for two nurses. The second
floor contains rooms for 19 nurses, and has similar accessories
to the first floor.
The building is of a very simple character, being of white
bricks with stone dressings. The staircases and all the
corridors are of fire-proof construction and the well of the
staircase contains a lift for luggage. The sitting-rooms are
warmed by Teale fireplaces, and hot-water radiators are
placed in the corridors. The whole building is lighted by
electricity.
The total accommodation is for 43 nurses.
The architects were Messrs. Young and Hall, of Southamp-
ton Street, Bloomsbury, and they seem to have gone carefully
into the subject, obtaining much room on small ground space.
The cost of the home without furnishing was ?8,365.
ROOF
FIRST FLOOR PLRN ?
SECOND FLOOR PLAN IS SIMILAR BUT CONTAINS
ROOMS FOR 19 NURSES.

				

## Figures and Tables

**Figure f1:**
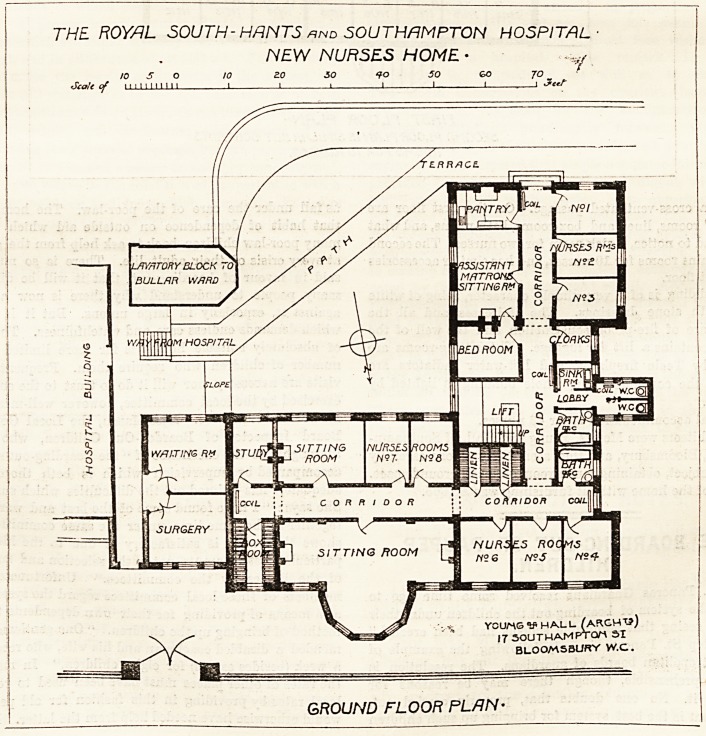


**Figure f2:**